# Characteristics of Eyes With Neovascular Age-Related Macular Degeneration Requiring Frequent Anti-vascular Endothelial Growth Factor Injections

**DOI:** 10.7759/cureus.50817

**Published:** 2023-12-20

**Authors:** Michiko Taketani, Hisaya Arakawa, Ichiro Maruko, Taiji Hasegawa, Tomohiro Iida

**Affiliations:** 1 Ophthalmology, Tokyo Women's Medical University, Tokyo, JPN

**Keywords:** dry macula, tachyphylaxis, non-responders, polypoidal choroidal vasculopathy, neovascular age-related macular degeneration

## Abstract

Objective

In this study, we aimed to determine the characteristics of neovascular age-related macular degeneration (AMD) patients requiring frequent anti-vascular endothelial growth factor (VEGF) therapy.

Methods

This was a retrospective observational study involving the review of 32 eyes of 31 patients (25 men and six women, mean age: 74.3 years) treated with anti-VEGF injections for less than eight weeks and at least one year of follow-up. The subtype of macular neovascularization (MNV), follow-up duration, number of injections, visual acuity, and exudative changes during the study period were evaluated.

Results

Twenty-nine eyes (90.6%) had MNV under the retinal pigment epithelium (RPE), including 11 eyes with type 1 MNV and 18 eyes with polypoidal choroidal vasculopathy (PCV). Only three eyes had type 2 MNV (9.4%) above the RPE. The mean follow-up period was 28.7 ± 16.5 months, and the mean number of injections was 21.5 ± 11.8. The mean visual acuity [logarithm of the minimum angle of resolution (logMAR) units] was 0.19 ± 0.23 at the initial visit to our hospital, which decreased non-significantly to 0.24 ± 0.4 at the final visit (p=0.63). The exudation in four eyes (two with type 1 MNV and two with PCV) never resolved. The exudation remained in 27 eyes (84%) even after every four weeks of treatment, and it was present in five eyes (16%) in the treatment interval of eight weeks.

Conclusions

In the eyes receiving frequent anti-VEGF injections, the sub-RPE MNV might have affected the response to the treatment. Although patients requiring frequent anti-VEGF therapy did not have a significant decrease in their visual acuity, 84% of the eyes had exudations even with monthly injections.

## Introduction

Intravitreal injections of anti-vascular endothelial growth factor (VEGF) are now established as the best treatment modality for macular neovascularization (MNV) secondary to age-related macular degeneration (AMD) [1-4]. However, as neovascular AMD is a chronic disease characterized by difficulty in maintaining visual acuity without continuous anti-VEGF injections, i.e., intravitreal aflibercept (IVA) or intravitreal ranibizumab (IVR), there has been much debate about the optimal dosing regimen for these patients. In general, the pro re nata (PRN) regimen [5], which consists of three consecutive monthly doses as a loading phase followed by treatment at the time of exudation, and the treat and extend (TAE) regimen [6-8], which comprises proactive treatment at two- or four-week intervals, are commonly used. However, some non-responders are resistant to anti-VEGF therapy, and cases of tachyphylaxis have been documented, where the gradual decrease of therapeutic efficacy follows the initial good response to treatment. We have found that a significant number of patients require monthly anti-VEGF injections.

A multicenter study, which included patients at our hospital as well, found that when IVA was administered to patients with neovascular AMD at four-week intervals by the TAE method with a maximum extension period of three months, 60% of the patients were subsequently treated at three-month intervals after two years, while 20% of the patients required monthly treatments [9]. These patients requiring monthly administration included cases of non-responders and tachyphylaxis [1-4], and they may need new treatment strategies such as photodynamic therapy (PDT) with verteporfin or the use of relatively new agents, i.e., brolucizumab or faricimab. However, the characteristics of patients who require frequent anti-VEGF therapy have not yet been determined. In light of this, this study aimed to determine the characteristics of patients requiring frequent anti-VEGF injections for neovascular AMD. To that end, we retrospectively reviewed the data of patients who underwent frequent anti-VEGF treatment.

## Materials and methods

We employed a retrospective study design, and the procedures used adhered to the tenets of the Declaration of Helsinki. The Institutional Review Board of Tokyo Women's Medical University School of Medicine approved the procedures used. All examinations were performed after obtaining signed informed consent from the patients.

Patients with neovascular AMD treated at the Department of Ophthalmology, Tokyo Women's Medical University from January to April 2020, with a treatment interval of less than eight weeks and with six or more injections in the past year or four or more injections within the last six months, were included. All patients were treated with IVA or IVR for at least one year, regardless of whether the initial treatment was performed in our department. In all cases, fluorescein angiography (FA) and indocyanine green angiography (IA) were performed with the HRA2 device (Heidelberg Engineering, Heidelberg, Germany) at the time of diagnosis of neovascular AMD, and typical AMD or polypoidal choroidal vasculopathy (PCV) was diagnosed in each case. Cases with retinal angiomatous proliferation as type 3 MNV were not included. Cases with typical AMD were classified as classic MNV or occult MNV based on the FA results, and they were determined as type 1 MNV if the lesion was located posterior to the retinal pigment epithelium (RPE) and as type 2 MNV if it was located anterior to the RPE on optical coherence tomography (OCT). The current study classified mixed type 1 and type 2 MNV as type 2 MNV. Polypoidal lesion in PCV was located at the sub-RPE. DRI-OCT (Topcon, Tokyo, Japan) was used to obtain the OCT images. Subfoveal choroidal thickness (SCT) was also measured in all cases at the first visit to our hospital. SCT was defined as the distance between Bruch’s membrane and the inner surface of the sclera at the fovea. Lesion size was measured as the greatest linear dimension (GLD) in all eyes, not just those that underwent PDT; GLD was measured in late FA with reference to IA findings by HRA2.

The following factors were also evaluated: history of anti-VEGF therapy at the previous hospital, follow-up period at our hospital, visual acuity at the initial and final visits, number of injections in the past, administration interval at the time of treatment, and history of PDT and drug switching therapy. In addition, ophthalmoscopic and OCT findings at the time of treatment were used to assess the presence of subretinal hemorrhage, intraretinal fluid (IRF), subretinal fluid (SRF), and serous pigment epithelial detachment (PED) greater than one disc diameter. In the current study, cases with an interval of less than eight weeks between treatments were classified as frequent treatment cases, and those who achieved dry macula at least once were classified as tachyphylaxis patients, while those who never achieved dry macula were classified as non-responder cases.

Statistical analysis

All tests to determine the significance of differences were two-tailed, and a p-value <0.05 was considered statistically significant. Mann-Whitney U tests were used to assess the significance of differences in means. All statistical analyses were performed using the free software EZR with the customization capabilities of R (The R Foundation for Statistical Computing, Vienna, Austria) [10].

## Results

Thirty-two eyes of 31 patients (25 men and six women, mean age: 74.3 years) were treated for less than eight weeks. The characteristics of eyes requiring frequent anti-VEGF injections for neovascular AMD are shown in Table 1.

**Table 1:** Characteristics of eyes requiring frequent treatments SD: standard deviation; BCVA: best-corrected visual acuity; logMAR: logarithm of the minimum angle of resolution; GLD: greatest linear dimension; PCV: polypoidal choroidal vasculopathy; MNV: macular neovascularization; IRF: intraretinal fluid; SRF: subretinal fluid; Serous PED: serous pigment epithelial detachment greater than one disc diameter

Characteristics		Values	P-value
Follow-up duration, months, mean ± SD		28.7 ± 16.5	
Number of injections, mean ± SD		21.5 ± 11.8	
BCVA, logMAR, mean ± SD	initial	0.19 ± 0.23	0.63
	final	0.24 ± 0.40	
GLD, µm, mean ± SD		2906 ± 1161	
PCV, n (%)		19 (59.4%)	
Type1 MNV, n (%)		11 (34.4%)	
Type 2 MNV, n (%)		2 (6.2%)	
Subretinal hemorrhage		8	
IRF		4	
SRF		31	
Serous PED		6	
Previous treatment, n		16	
Photodynamic therapy, n		10	
Switch and switch-back, n		6	
Final intervals, weeks, mean ± SD		5.5 ± 1.7	

Of note, 50% (16 eyes) of the patients had already been treated with anti-VEGF therapy at their previous hospital and referred to our institution as treatment-refractory patients. The mean follow-up at our hospital was 28.7 ± 16.5 months (range: 6-66 months) and started with IVA. The mean best corrected visual acuity (BCVA) decreased slightly from 0.19 ± 0.23 logarithm of the minimum angle of resolution (logMAR) units at baseline to 0.24 ± 0.40 logMAR units at the final visit (p=0.63). The mean number of injections at our hospital was 21.5 ± 11.8 (range: 6-48), and the mean interval between injections at the time of injection was 5.5 ± 1.7 weeks. Ten eyes were also treated with PDT, and six eyes were treated with switch therapy by IVR. Subretinal hemorrhages were observed in eight eyes (five of PCV, one of type 1 MNV, and two of type 2 MNV). While most of the eyes (31 eyes) had the SRF, only four eyes had the IRF. 

The exudation in four eyes (two eyes with type 1 MNV and two eyes with PCV) never resolved during the follow-up period. Two of the four eyes developed subfoveal hemorrhage and significant vision loss. As for the remaining two eyes, one eye improved and one eye remained unchanged. Exudation remained in 27 eyes (84%) after every four weeks of treatment and was present in five eyes (16%) during the treatment interval with the last visit at eight weeks.

**Figure 1:**
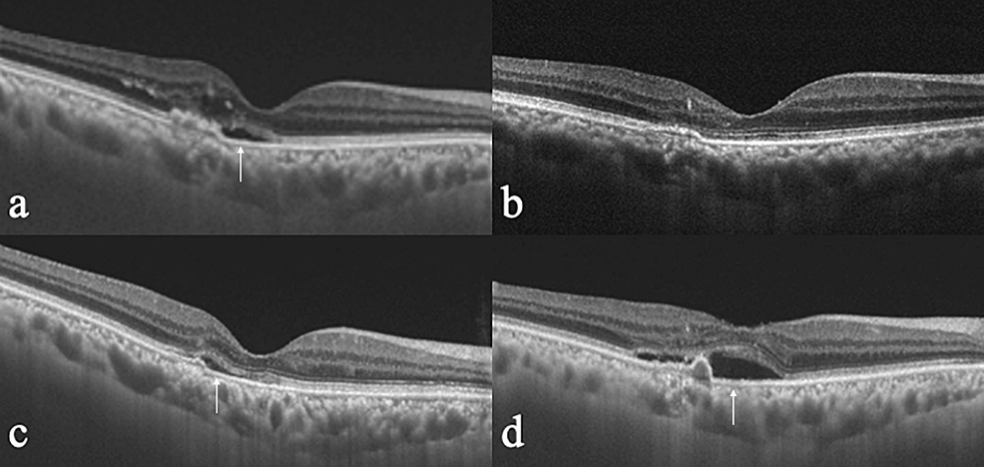
Tachyphylaxis case worsens after two-month interval of injection The patient was an 81-year-old man. Optical coherence tomographic (OCT) images in eyes require frequent anti-vascular endothelial growth factor treatment during the follow-up period a: At the baseline. b: Dry macula at three months. c: Wet macula at 24 months. d: Wet macula at 26 months. White arrows indicate the presence of subretinal fluid

Among the neovascular AMD types, PCV accounted for 59.4% (19 eyes), and typical AMD for 40.6% (13 eyes) of all eyes (11 eyes with type 1 MNV and two eyes with type 2 MNV). Thus, 30 eyes (93.8%) had sub-RPE MNV. Only four eyes had single polypoidal lesions, while multiple polypoidal lesions were observed in the other 15 eyes. Of all 32 eyes, 87.5% (28 eyes) had tachyphylaxis, including 17 eyes with PCV and 11 eyes with typical AMD (nine with type 1 MNV and two with type 2 MNV). The number of non-responders accounted for 12.5% (four eyes), including two PCV and two type 1 MNV. The findings in a representative case of tachyphylaxis are shown in Figure 1.

Sixteen eyes of 16 patients (14 males and two females, mean age: 75.1 years) treated at our hospital from the beginning itself included 10 eyes with PCV (62.5%), five eyes with type 1 MNV (31.3%), and one eye with type 2 MNV (8.3%). The mean follow-up in our department was 33.7 ± 18.4 months. The mean number of injections in our clinic was 22.5 ± 13.2, and the mean interval between injections at the time of injection was 5.4 ± 1.9 weeks. There was no significant difference in age (p=0.65), follow-up period (p=0.12), number of injections (p=0.79), and interval between injections (p=0.64) compared to the eyes that had started treatment at the previous clinic. The mean SCT in eyes with treatment initiated at our hospital was 285 ± 109 µm, which was thicker than the 226 ± 115 µm in eyes with prior treatment at the previous hospital; this difference did not reach statistical significance (p=0.07).

## Discussion

Neovascular AMD eyes with PCV and type 1 MNV constituted the most common cases requiring frequent anti-VEGF therapy, and the most common exudative finding was SRF. Eyes with these findings did not have a significant reduction in BCVA. Of note, 87.5% of the eyes had a dry macula at least once during the follow-up period and then had a recurrence of exudation. These eyes were classified as tachyphylaxis eyes. The remaining 12.5% were non-responders who never had a resolution of exudation.

There have been several reports of anti-VEGF-tolerant AMD cases in the literature. Non-responders were reported in 10-20% of those treated with ranibizumab [11], 5% with aflibercept [12], and tachyphylaxis was reported in 8.9% with aflibercept [13]. Regarding the subtype of neovascular AMD, refractory to anti-VEGF therapy in PCV has been reported for some time, although the number of cases is not substantial [14,15]. Refractory cases of occult type MNV, now type 1 MNV, have also been reported [16,17]. However, PCV is more common in Asians and less common in Caucasians, and there are few reports including both PCV and type 1 MNV as refractory cases. The baseline data of a paper from Japan on tachyphylaxis cases stated that PCV and type 1 MNV were the most common refractory cases; in addition, these eyes were characterized not only by sub-RPE lesions but also by exudative findings, mainly SRF without IRF [18].

Currently, there is a common approach to evaluate the effect of exudative findings on BCVA based on the anatomical categories of IRF, SRF, and PED. In a post hoc analysis of the Comparisons of Age-Related Macular Degeneration Treatments Trials (CATT) study, which compared the effects on BCVA among four groups of patients, one receiving a fixed monthly dose of ranibizumab or bevacizumab and the other receiving a PRN dose of ranibizumab or bevacizumab with monthly visits [19,20], the two-year [19] and five-year [20] results showed that eyes with SRF within 1 mm of the fovea had better visual acuity than eyes with SRF away from the fovea. A post hoc analysis of the VIEW study, a large clinical trial of IVA, also showed that patients with SRF had better visual acuity than patients without SRF [21]. These results suggest that the visual prognosis improves when the SRF is persistent. The two-year results of the FLUID study, which prospectively compared the effects of intensive treatment in eyes with SRF (SRF at fovea <200 µm), showed no difference in final visual acuity [22]. Our results support these findings; some SRF does not worsen visual acuity for at least two years. This could explain why the eyes with SRF in our study did not show a significant decrease in BCVA despite frequent anti-VEGF treatment.

A previous multicenter study, including patients at our hospital, reported the two-year results of aflibercept administered by TAE at monthly intervals [9]. The results showed that approximately 60% of the eyes were treated every three months after two years, while 20% of the eyes required monthly injections. Interestingly, the group requiring monthly injections tended to have better visual acuity than those treated every three months. This result suggests that monthly injections are not a negative sign for vision maintenance alone, disregarding the burden of frequent hospital visits, financial expenses, and risks of intraocular injections. It has also been reported that fluctuations in the amount of SRF or IRF may cause vision loss [23]. The eyes that required frequent injections without complete resolution of exudation could avoid vision deterioration by stabilizing disease activity.

On the other hand, in the post hoc analysis of the FLUID study, when the relationship between changes in visual acuity and the amount of SRF was evaluated, the presence of SRF in the range of 1-6 mm and not including the central fovea had a negative correlation with visual acuity [24]. This suggests that the presence of SRF is a factor that affects vision to some extent. Two of the four eyes in the current study that never had exudation resolved developed subretinal hemorrhage and significantly reduced vision loss. Undoubtedly, persistent exudation of SRF and IRF over a long period is a risk for vision loss. Therefore, new therapeutic strategies are needed for such cases. Hara et al. [18] performed PDT with anti-VEGF therapy in 15 of 28 eyes with tachyphylaxis to aflibercept and found that exudation resolved in 13 eyes (87%) at one month and in five eyes (38%) at six months. However, there were some problems with PDT, such as subretinal hemorrhage, future RPE atrophy, and inability to perform frequent treatments. In a large randomized clinical trial, brolucizumab and faricimab were not found inferior to aflibercept in visual acuity [25,26]. These relatively new anti-VEGF agents are expected to be effective for switch therapy in non-responders and tachyphylaxis cases [27-29].

This study has several limitations, including the small sample size and short follow-up period. In addition, half of the patients in this study had been treated with anti-VEGF injections at their previous clinic. However, there was no significant difference in any factors between those with and without prior treatment. Nowadays, anti-VEGF therapy is more common and considered an easy-to-treat method for MNVs, even in small clinics. The results of this study should help clinicians refer patients with uncontrollable exudation to a retinal specialist as early as possible. This study did not focus specifically on pachychoroid neovasculopathy. Recently, it has been pointed out that pachychoroid neovasculopathy can be confused with AMD and that the response to anti-VEGF therapy is different from that of AMD [30]. In the current study, SCT was measured, and although the choroid was slightly thicker in eyes treated at our hospital initially, there was no significant difference. Prospective studies with a larger number of patients are necessary to elucidate the extent of choroidal involvement in the pathogenesis of the disease.

## Conclusions

We believe the results of this study will help clinicians better understand the characteristics of non-responders and tachyphylaxis cases. The fact that most patients requiring frequent injections were tachyphylaxis patients suggests that subfoveal MNV itself is potentially resistant to anti-VEGF therapy. It is unknown whether this state can be maintained with frequent injections, and it will be necessary to reconsider the treatment regimen for such patients in the future, for instance, by employing a combination of PDT or drug modification.
